# Effects of fasting on serial measurements of hyperpolarized [1‐^13^C]pyruvate metabolism in tumors

**DOI:** 10.1002/nbm.3568

**Published:** 2016-06-16

**Authors:** Eva M. Serrao, Tiago B. Rodrigues, Ferdia A. Gallagher, Mikko I. Kettunen, Brett W. C. Kennedy, Sarah L. Vowler, Keith A. Burling, Kevin M. Brindle

**Affiliations:** ^1^ Cancer Research UK Cambridge InstituteUniversity of Cambridge, Li Ka Shing CentreCambridgeUK; ^2^ Department of BiochemistryUniversity of CambridgeCambridgeUK; ^3^ Department of RadiologyUniversity of Cambridge, Cambridge Biomedical CampusCambridgeUK; ^4^ A. I. Virtanen Institute for Molecular SciencesUniversity of Eastern FinlandKuopioFinland; ^5^ Core Biochemical Assay LaboratoryCambridge University Hospitals NHS Foundation TrustCambridgeUK

**Keywords:** repeatability, hyperpolarization, fasting, pyruvate, lymphoma, cancer

## Abstract

Imaging of the metabolism of hyperpolarized [1‐^13^C]pyruvate has shown considerable promise in preclinical studies in oncology, particularly for the assessment of early treatment response. The repeatability of measurements of ^13^C label exchange between pyruvate and lactate was determined in a murine lymphoma model in fasted and non‐fasted animals. The fasted state showed lower intra‐individual variability, although the [1‐^13^C]lactate/[1‐^13^C]pyruvate signal ratio was significantly greater in fasted than in non‐fasted mice, which may be explained by the higher tumor lactate concentrations in fasted animals. These results indicate that the fasted state may be preferable for the measurement of ^13^C label exchange between pyruvate and lactate, as it reduces the variability and therefore should make it easier to detect the effects of therapy. © 2016 The Authors. *NMR in Biomedicine* published by John Wiley & Sons Ltd.

**Abbreviations used**
*ALT*: *alanine aminotransferase*
*ANOVA*: *analysis of variance*
*BA*: *Bland*–*Altman*
*CCC*: *concordance correlation coefficient*
*COV*: *coefficient of variation*
*DCE*: *dynamic contrast‐enhanced*
*DTPA*: *diethylenetriaminepentaacetic acid*
*EDTA*: *ethylenediaminetetraacetic acid*
*FLASH*: *fast low‐angle shot*
*HEPES*: *4‐*(*2‐hydroxyethyl*)*‐1‐piperazineethanesulfonic acid*
^*18*^*F‐FDG‐PET*: ^*18*^ *F* *‐fluorodeoxyglucose positron emission tomography*
*k*_*P*_: *apparent exchange rate constant for conversion of pyruvate to lactate*
*Lac*: *lactate*
*LDH*: *lactate dehydrogenase*
*PDH*: *pyruvate dehydrogenase*
*Pyr*: *pyruvate*
*ROI*: *region of interest*
*TSP*: *3‐*(*trimethylsilyl*)*‐2*,*2*′,*3*,*3*′*‐tetradeuteropropionic acid*

## Introduction

The introduction of hyperpolarized ^13^C‐labeled cell substrates has created a renewed interest in the field of MRS by allowing the real‐time assessment of tissue metabolism *in vivo* 1. [1‐^13^C]Pyruvate ([1‐^13^C]Pyr) is the most well‐studied substrate, having shown promise in preclinical studies in oncology, particularly as an imaging marker for the detection of early response to therapy and as a possible marker for tumor grading and early lesion detection 2, 3, 4, 5. Pyr has also translated to the clinic, with a study in prostate cancer 6. As the end product of glycolysis, Pyr can be reduced reversibly by NADH to generate lactate (Lac), in a reaction catalyzed by lactate dehydrogenase (LDH), or transaminated by glutamate to form alanine, in a reaction catalyzed by alanine aminotransferase (ALT). Irreversible decarboxylation of Pyr to carbon dioxide, in a reaction catalyzed by mitochondrial pyruvate dehydrogenase (PDH), may also occur and, in some tissues, is sufficiently fast to give observable labeling of carbon dioxide and bicarbonate with a hyperpolarized ^13^C label 7. Data from hyperpolarized ^13^C MRSI measurements are often presented as either signal ratios 8 or apparent exchange rates when the metabolite signals are analyzed as a function of time. Both measurements are affected by Pyr delivery to the tumor, tumor cell uptake and subsequent enzyme‐catalyzed exchange 2, 9, 10, 11, 12.

Despite extensive preclinical use of hyperpolarized [1‐^13^C]Pyr, with promising future clinical applications in oncology 2, 13, 14, notably in the serial assessment of treatment response, there is a lack of information regarding the repeatability of these measurements. The determination of repeatability is important for an understanding of whether the observed changes in kinetics of hyperpolarized [1‐^13^C]Pyr conversion to Lac reflect real effects of therapy or, rather, changes in physiology and/or method variability.

^18^F‐Fluorodeoxyglucose positron emission tomography (^18^F‐FDG‐PET), similarly to hyperpolarized [1‐^13^C]Pyr, also assesses an aspect of glycolysis. Several studies have evaluated the repeatability of ^18^F‐FDG‐PET, as well as the main factors underlying the observed variability. A major factor affecting ^18^F‐FDG uptake is high blood glucose levels 15, as a result of competition between glucose and ^18^F‐FDG for transport into the cell and subsequent phosphorylation. Therefore, it is recommended that, for ^18^F‐FDG‐PET examinations, there is some control over blood glucose levels, either by overnight fasting 16 or by the measurement of blood glucose levels at the time of injection 17.

We have investigated here whether the variability and repeatability of measurements with hyperpolarized [1‐^13^C]Pyr are also affected by fasting.

## Materials and Methods

### Tumor implantation and animal preparation

Tumors were established by subcutaneous inoculation of a suspension of 5 × 10^6^ EL4 murine lymphoma cells (in a volume of 100 μL) in the left flank of 8–10‐week‐old C57/Blk6 female mice. Tumors were allowed to grow for 10 days (volume, ~2 cm^3^; maximum diameter, 1.5 cm), after which the mice were divided into two cohorts: fasted and non‐fasted. Fasted mice were deprived of food for 18 h prior to the first ^13^C spectroscopic examination. Experiments were conducted in accordance with project and personal licenses issued under the United Kingdom Animals (Scientific Procedures) Act, 1986. Protocols were approved by the Cancer Research UK, Cambridge Institute Animal Welfare and Ethical Review Body.

### MRI/MRS protocols

Tumor‐bearing mice were anesthetized initially by inhalation of a mixture of O_2_ (0.3 L/min) and air (0.9 L/min) containing 3% isoflurane (Isoflo, Abbotts Laboratories Ltd, Maidenhead, UK) and were maintained during the MRS experiment with 1–2% isoflurane in O_2_ and air, which was delivered via a facemask. Animals were taped into a cradle to minimize breathing‐related motion, and placed in a heated MR probe, which maintained the core body temperature at ~37 °C (monitored by a rectal probe). Respiratory rate and body temperature were monitored during the experiment using a Biotrig physiological monitor (Small Animal Instruments, Stony Brook, NY, USA). A cannula was inserted into a tail vein and its patency was maintained through the use of heparin diluted in sterile physiological saline (100 U/mL). A 20‐mm‐diameter curved surface coil (Rapid Biomedical GmbH, Rimpar, Germany) was placed over the tumor. The entire assembly was placed in a ^13^C/^1^H volume coil (Rapid Biomedical GmbH) in a 7‐T horizontal bore magnet (Agilent, Palo Alto, CA, USA). The tumor was localized using transverse ^1^H images acquired using a spin‐echo pulse sequence (TR = 1.5 s; TE = 10 ms; field of view, 40 mm × 40 mm; data matrix, 128 × 128; slice thickness, 2 mm; 15 slices).

### [1‐^13^C]Pyr hyperpolarization

Pyr was hyperpolarized as described previously 1. Briefly, [1‐^13^C]pyruvic acid samples (44 mg, 14 mol/L; 91% ^13^C), containing 15 mmol/L of trityl radical, tris(8‐carboxy‐2,2,6,6‐tetra‐(hydroxyethyl)‐benzo‐1, 2, 3, 4, 5‐bis‐1, 3‐dithiole‐4‐yl)‐methyl sodium salt (OX063; GE Healthcare, Amersham, Buckinghamshire, UK) and 1.5 mmol/L gadolinium chelate (Dotarem), were polarized using a microwave source at 93.982 GHz and 100 mW for 1 h in a 3.35‐T Hypersense (Oxford Instruments Molecular Biotools Ltd., Abingdon, Oxfordshire, UK) polarizer. The frozen sample was then dissolved in a solution containing 40 mm 4‐(2‐hydroxyethyl)‐1‐piperazineethanesulfonic acid (HEPES), 94 mm NaOH, 30 mm NaCl and 50 mg/L ethylenediaminetetraacetic acid (EDTA) at ~180 °C and ~1 MPa. Polarization levels ranged from 16% to 25%, measured using a bench‐top polarimeter (^13^C‐MQC polarimeter, Oxford Instruments Molecular Biotools Ltd., Abingdon, Oxfordshire, UK).

### NMR spectroscopy of tumors

**Figure 1:**
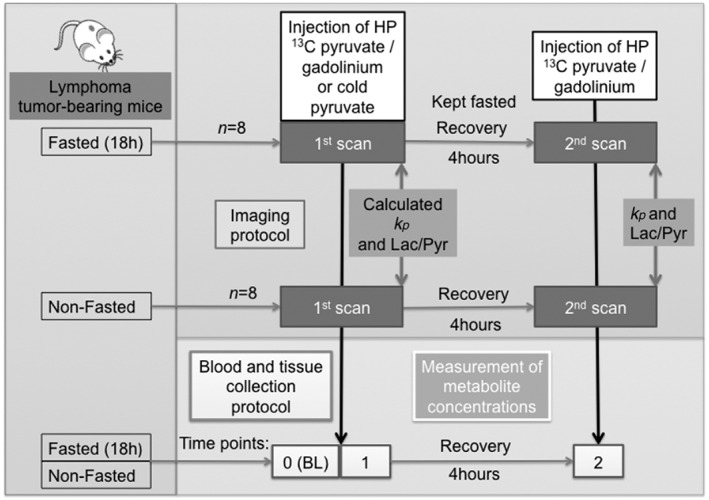
Schematic representation of study design. EL4 tumor‐bearing mice were randomly divided into two groups: fasted for 18 h and non‐fasted. Mice then underwent the same imaging protocol twice in the same day, with a 4‐h recovery period between examinations, in which the non‐fasted mice were given access to food. The first and second ^13^C spectroscopic examinations are referred to as the 1st and 2nd scans, respectively. The tumor [1‐^13^C]lactate/[1‐^13^C]pyruvate ([1‐^13^C]Lac/[1‐^13^C]Pyr) signal ratio, at 30 s after injection of hyperpolarized (HP) [1‐^13^C]Pyr, and *k*_P_ were calculated from the time course of pyruvate (Pyr) and lactate (Lac) labeling. In another group of animals, the ratio of the area under the contrast agent concentration curve (AUC) in the tumor and adjacent muscle was calculated after injection of contrast agent at each of the two time points, with the mice at the second time point having had an injection of unlabeled, non‐HP, Pyr at the first time point. A different cohort of mice, prepared in the same manner, was sacrificed at three different time points: baseline (BL, 0) (no injection of Pyr), 1 (after injection of Pyr) and 2 (after two injections of Pyr, 4 h apart), with each mouse submitted to at least 30 min of anesthesia, which is the time for which the animals were anesthetized for the HP [1‐^13^C]Pyr studies.

From the total number of animals (*n* = 30), paired data were only obtained from 16 animals, as the remainder showed a poor signal‐to‐noise ratio in the ^13^C spectra from one of the examinations, reflecting the difficulty in obtaining good sequential tail vein injections over such a relatively short period of time. Paired ^13^C spectroscopic data, acquired 4 h apart, were obtained from eight fasted and eight non‐fasted animals (Table 1). The mice were examined on the same day as the tumor model showed rapid growth 18, 19, which could alter the metabolic status of the tumor if the animals were examined on subsequent days. After the first examination, mice were recovered from anesthesia and food was provided only to the non‐fasted group (Fig. 1).

**Table 1:** Mean coefficient of variation of *k*_P_ and the hyperpolarized [1‐^13^C]lactate/[1‐^13^C]pyruvate signal ratio in fasted and non‐fasted tumor‐bearing mice following repeated measurements

HP [1‐^13^C]pyruvate	*n* (mice)	CCC	Mean	SD	COV (%)
		(95% CI)			

Fasted					
*k*_P_	8	0.89 (0.60–0.97)	0.141	0.016	11.773*
Lac/Pyr	8	0.83 (0.44–0.95)	5.671	0.942	14.998

Non‐fasted					
*k*_P_	8	0.62 (0.03–0.89)	0.1	0.021	24.767
Lac/Pyr	8	0.47 (−0.14–0.82)	3.563	0.934	29.499

Fasted		*n*		*k*_P_	Lac/Pyr
1st measurement		11		0.137 ± 0.05	5.49 ± 2.23**
2nd measurement		10		0.138 ± 0.06	5.41 ± 3.04

Non‐fasted		*n*		*k*_P_	Lac/Pyr
1st measurement		17		0.108 ± 0.04	3.67 ± 1.7
2nd measurement		8		0.107 ± 0.03	3.89 ± 1.25

CCC, concordance correlation coefficient; CI, confidence interval; COV, coefficient of variation; HP, hyperpolarized; *k*_P_, apparent exchange rate constant of conversion of pyruvate to lactate; Lac, lactate; Pyr, pyruvate; SD, standard deviation.

*k*_P_ and [1‐^13^C]Lac/[1‐^13^C]Pyr signal ratios were calculated from the time courses of ^13^C labeling in lactate and pyruvate in tumors in fasted and non‐fasted mice, acquired 4 h apart. The mean, SD, intra‐individual COV and CCC were calculated from 16 measurements for both the fasted and non‐fasted animals.

* *p* = 0.01, significantly different from non‐fasted animals

** *p* = 0.02, significantly different from non‐fasted animals

Following intravenous injection of hyperpolarized [1‐^13^C]Pyr (10 mL/kg, 82 mm), single transient spectra from a 6‐mm‐thick slice through the tumor were acquired using a slice‐selective excitation pulse with a nominal flip angle of 10°. One hundred and twenty ^13^C spectra were acquired every 2 s beginning 10 s after injection. The processing of ^13^C spectroscopic data was performed in Matlab (Mathworks, Massachusetts, USA). The integrated peak intensities of hyperpolarized [1‐^13^C]Pyr and [1‐^13^C]Lac were fitted to the modified Bloch equation for two‐site exchange to calculate the rate constants *k*_L_ and *k*_P_ and the apparent spin–lattice relaxation rates, as described previously 2.

(1) $$L\;\frac{\underset{\leftarrow }{k_{P}}}{\vec{k_{L}}}\;P$$

(2) $$\frac{{dL}_{Z}}{dt}=-R_{1,L}\left(L_{Z\;}–L_{\infty }\right)+k_{P}P_{Z}-k_{L}L_{Z}$$

(3) $$\frac{{dP}_{Z}}{dt}=-R_{1,P}\left(P_{Z\;}–P_{\infty }\right)+k_{L}L_{Z}-k_{P}P_{Z}$$

where *L*_*Z*_ and *P*_*Z*_ are the *z* magnetizations of the ^13^C nucleus in the Lac and Pyr carboxyl carbons, respectively, *R*_1,L_ and *R*_1,P_ are the spin–lattice relaxation rates (1/*T*_1_), and *L*_∞_ and *P*_∞_ are the equilibrium magnetizations of Lac and Pyr, respectively, which are effectively equivalent to their concentrations. The relaxation rates for Lac and Pyr were assumed to be the same. We have shown previously that this assumption has little effect on the fitted rate constants [see supplementary fig. 2 in ref. 2]. *L*_∞_/*P*_∞_ was obtained from the ratio of the fitted rate constants. The ^13^C‐labeled Lac/Pyr ratios were calculated from *L*_*Z*_/*P*_*Z*_ at 30 s.

**Figure 2:**
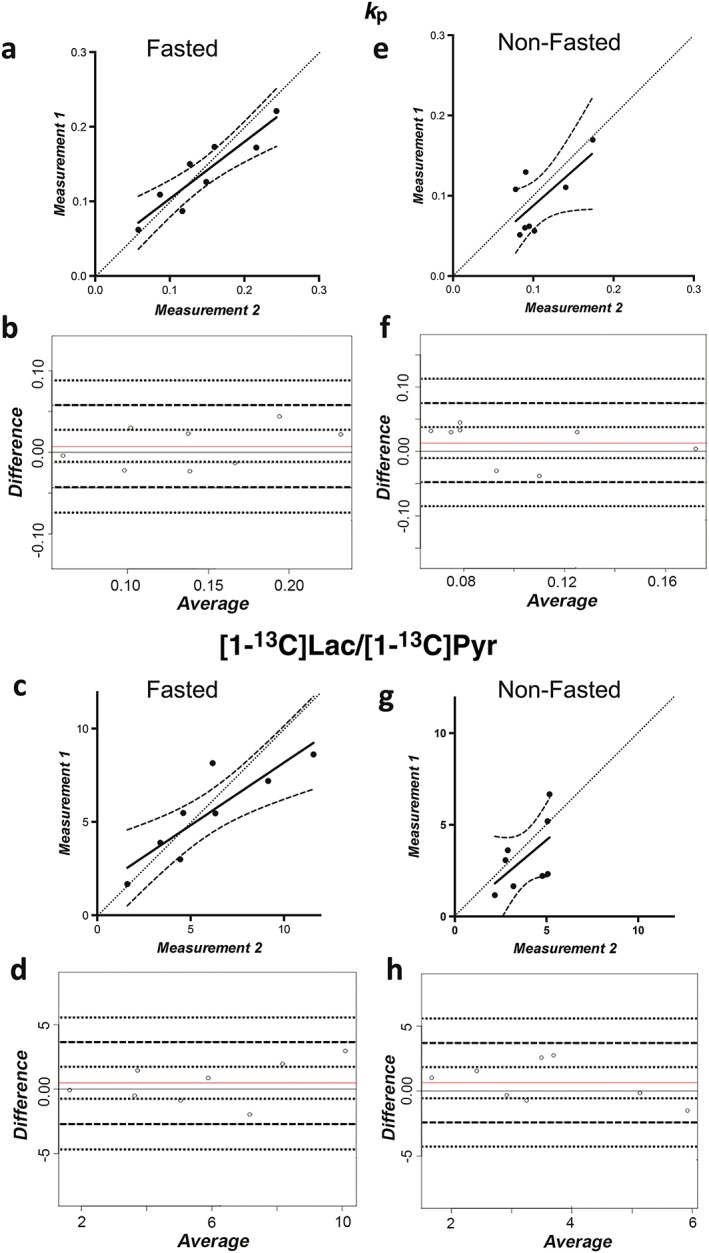
Repeatability of *k*_P_ and [1‐^13^C]lactate/[1‐^13^C]pyruvate ([1‐^13^C]Lac/[1‐^13^C]Pyr) signal ratio measurements in fasted mice (a–d) and non‐fasted mice (e–h) (see Fig. 1). Measurements in a single animal from the 1st scan (measurement 1) are plotted against measurements from the same animal from the 2nd scan (measurement 2). The full line indicates the best‐fit line between these two measurements (a & c, e & g). The broken lines indicate the 95% confidence limits of the best‐fit line. The dotted lines represent perfect agreement between the two measurements. For each mouse, the differences in *k*_P_ between the 1st and 2nd scans are plotted against the average of *k*_P_ from the 1st and 2nd scans (b & f). This Bland–Altman (BA) plot 23 was used to test the assumption of constant variances across the differences in *k*_P_ *.* The full line at 0.00 is that of perfect agreement between the measurements of *k*_P_ from the 1st and 2nd scans, and the full line above is the mean difference of *k*_P_ between the 1st and 2nd scans. The broken lines indicate the 95% confidence limits of agreement. The dotted lines represent the 95% confidence intervals for these limits of agreement. The BA plots for the [1‐^13^C]Lac/[1‐^13^C]Pyr ratios are shown in (d & h).

### Dynamic contrast‐enhanced MRI (DCE‐MRI)

Separate cohorts of mice, fasted (*n* = 7) and non‐fasted (*n* = 7), were used for DCE‐MRI measurements of tumor perfusion, in which the mice were injected with Gd^3^^+^‐DTPA (diethylenetriaminepentaacetic acid; 200 mmol/kg; Magnevist, Bayer Schering Pharma, Leverkusen, Germany) (Table 2). The imaging protocol simulated the protocol used for the ^13^C hyperpolarized measurements. Briefly, for each cohort (seven fasted, seven non‐fasted), four mice were injected with contrast agent (1st scan) and three mice were injected with unlabeled Pyr (82 mm, 10 mL/kg), followed by injection of contrast agent 4 h later (2nd scan) (Fig. 1). Before each injection, animals were submitted to at least 30 min of anesthesia, which is the same time for which they were anesthetized for the hyperpolarized [1‐^13^C]Pyr measurements.

**Table 2:** Measurements of tumor perfusion using dynamic contrast agent‐enhanced ^1^H MRI

AUC (tumor/muscle)			
Fasted 1st scan	Fasted 2nd scan	Non‐fasted 1st scan	Non‐fasted 2nd scan
6.8 ± 2.8	9.7 ± 4.7	12.7 ± 5.6	5.9 ± 3.0

Tumor/muscle (thigh) ratio for the area under the contrast agent concentration curve (AUC) at 45 s after injection of contrast agent for fasted and non‐fasted EL4 tumor‐bearing mice. Acquisitions were performed at the same time points as for the hyperpolarized [1‐^13^C]pyruvate study, with mice from Group 2 having received a dose of unlabeled non‐polarized pyruvate (10 mL/kg, 82 mm) and 30 min of anesthesia 4 h before the contrast agent‐enhanced ^1^H images were acquired. Mean ± standard error of the mean.

DCE‐MRI data were acquired using a *T*_1_‐weighted spin‐echo pulse sequence, as described previously 12. Briefly, first an inversion recovery fast low‐angle shot (FLASH) pulse sequence was used to measure the native spin–lattice relaxation rates (*R*_1_ = 1/*T*_1_). These inversion recovery data were fitted, pixel‐by‐pixel, to a mono‐exponential function to obtain a pre‐contrast *R*_1_ map. A dynamic *T*_1_‐weighted spin‐echo pulse sequence was then used to collect baseline images (6 or 10) prior to injection of contrast agent. Diluted Gd^3^^+^‐DTPA in sterile saline (0.9% sodium chloride) was then injected as a bolus through a tail vein catheter over 2–3 s and dynamic *T*_1_‐weighted spin‐echo images were acquired for 10 min post‐injection. These images were then converted to *R*_1_ relaxation rate maps, as described previously 12. Gd^3^^+^‐DTPA concentration curves were determined for all tumor‐containing slices with a manually delineated region of interest (ROI) in the tumor and also in adjacent thigh muscle. The area under the uptake curve up to 45 s (AUC 45) was calculated for each ROI.

### Blood and tissue collection and analysis

In a separate set of experiments (Table 3), tumor‐bearing mice (*n* = 24) were divided into two groups: fasted (*n* = 12) and non‐fasted (*n* = 12). In each group, the mice were further divided into three subgroups: Group 0, anesthetized for 30 min; Group 1, 30 min of anesthesia plus a bolus injection of 10 mL/kg of 82 mm unlabeled Pyr; Group 2, same as Group 1, plus a 4‐h recovery period, a second period of anesthesia for 30 min and another injection of 10 mL/kg of 82 mm unlabeled Pyr (Fig. 1). The timing of anesthesia and Pyr injection was the same as for the ^13^C MRS data acquisition protocol (Fig. 1). Blood was collected rapidly by cardiac puncture in all mice from each group (starting at 25 s after Pyr injection) and the tumors were quickly excised and freeze‐clamped 30 s after Pyr injection.

**Table 3:** Blood and tumor metabolite concentrations and excised tumor and mouse weights in fasted and non‐fasted mice

Sample	Metabolite	Groups (mean ± SD)					
		Fasted (baseline)	Fasted 1	Fasted 2	Non‐fasted (baseline)	Non‐fasted 1	Non‐fasted 2
Tumor concentration	Lactate (μmol/g wet tissue)		19.1 ± 2.7 (*n* = 3)*, ^a^	15.5 ± 2.0 (*n* = 3)		10.8 ± 4.1 (*n* = 3)	15.8 ± 5.9 (*n* = 3)
Tumor concentration	Alanine (μmol/g wet tissue)		3.9 ± 1.4 (*n* = 3)	2.9 ± 1.1 (*n* = 3)		2.6 ± 0.9 (*n* = 3)	5.3 ± 1.5 (*n* = 3)
Blood concentration	Lactate (mmol/L)	10.2 ± 2.9 (*n* = 5)	10.1 ± 2.6 (*n* = 3)	13.0 ± 1.8 (*n* = 4)	8.7 ± 0.8 (*n* = 5)	8.9 ± 1.4 (*n* = 3)	11.9 ± 1.3 (*n* = 4)
Blood concentration	Pyruvate (μmol/L)	140 ± 45 (*n* = 5)	193 ± 35 (*n* = 3)*, ^b^	183 ± 30 (*n* = 4)	110 ± 14 (*n* = 5)	128 ± 19 (*n* = 3)	135 ± 29 (*n* = 4)
Mouse weight (g)		20.0 ± 0.9 (*n* = 5)	23.7 ± 1.7 (*n* = 3)	20.3 ± 0.8 (*n* = 4)	25.3 ± 0.6 (*n* = 5)*, c, d	25.4 ± 3.9 (*n* = 3)	24.4 ± 1.7 (*n* = 4)
Tumor weight (g)			0.8 ± 0.5 (*n* = 3)	0.9 ± 0.6 (*n* = 3)		0.7 ± 0.1 (*n* = 3)	1.3 ± 0.3 (*n* = 3)

Blood metabolite concentrations were determined using spectrophotometric assays, and tumor alanine and lactate concentrations were measured by ^1^H NMR. Group 0 (baseline), anesthetized for 30 min; Group 1, 30 min of anesthesia plus a bolus injection of 10 mL/kg of 82 mm unlabeled pyruvate; Group 2, same as Group 1, plus a 4‐h recovery period, a second period of anesthesia for 30 min and another injection of 10 mL/kg of 82 mm unlabeled pyruvate. Mean ± standard deviation;

* *p* < 0.05.

a,b Significantly different from non‐fasted 1.

c Significantly different from fasted baseline.

d Significantly different from fasted 2.

Blood samples were centrifuged (18 800 ***g***) at 4 °C for 5 min in heparin‐coated Eppendorf tubes (~5 μL of 1000 U/mL). Pyr was analyzed using a colorimetric assay kit (AbCam, Cambridge, UK) and Lac by an enzymatic assay kit (Siemens Healthcare, Sudbury, UK), using a Siemens Dimension RxL analyzer. Perchloric acid extracts were prepared from the tumor tissues of Groups 1 and 2 for both fasted (*n* = 6) and non‐fasted (*n* = 6) mice using ice‐cold 7% perchloric acid (1: 8 w/v), which were then neutralized with KOH, lyophilized and dissolved in 99.9% deuterium oxide. High‐resolution ^1^H NMR spectra of plasma and tumor extracts were obtained at 14.1 T (25 °C, pH 7.2) in a Bruker 600‐MHz NMR spectrometer (Bruker, Ettlingen, Germany) using a 5‐mm probe. The acquisition conditions were as follows: 90° pulses; spectral width, 7.3 kHz; acquisition time, 4.5 s; 32 k data points; 64 transients; recycling time, 12.5 s. Chemical shifts were referenced to 3‐(trimethylsilyl)‐2,2′,3,3′‐tetradeuteropropionic acid (TSP) at 0.0 ppm, which was added to the sample at a concentration of 5 mm. Data were analyzed using ACDSpecManager (ACD/Labs, Bracknell, UK). The free induction decays were zero‐filled twice and multiplied by an exponential function prior to Fourier transformation. Peak integrals were normalized to the integral of the TSP resonance.

### Statistical analysis

Data were analyzed using SPSS (v21, IBM SPSS, Chicago, IL, USA) and GraphPad Prism v6 (GraphPad Software, San Diego, USA). The repeatability of measurements of *k*_P_ and the [1‐^13^C]Lac/[1‐^13^C]Pyr signal ratio, which was measured at 30 s after injection, were assessed from the two imaging examinations performed 4 h apart. Scatter plots (Measurement 1 *versus* Measurement 2) and Bland–Altman plots (difference *versus* mean) were generated. The concordance correlation coefficient (CCC), within‐subject (intra‐individual) standard deviation (SD) and coefficient of variation (COV) were calculated as described in refs. 20, 21. The COVs of *k*_P_ and the [1‐^13^C]Lac/[1‐^13^C]Pyr signal ratio were calculated, for each parameter, as the SD divided by the parameter mean, and expressed as a percentage. This was calculated for each individual and then averaged. COV can be used as a statistical measure of reliability 22 and has been used previously to assess reliability in FDG‐PET studies 21. Data were reported as mean ± SD, unless stated otherwise. Statistical significance was tested with Prism using a two‐tailed Student's *t*‐test or analysis of variance (ANOVA) (*post‐hoc* test: Tukey) when appropriate. Paired *t*‐tests were used when comparing paired data from the same mouse. The results were considered to be significant when *p* < 0.05.

## Results

### Repeatability of ^13^C MRS measurements with hyperpolarized [1‐^13^C]Pyr

Intravenous injection of hyperpolarized [1‐^13^C]Pyr (10 mL/kg, 82 mm) resulted in tumor signals from [1‐^13^C]Pyr and [1‐^13^C]Lac that were similar in intensity to those observed previously in this tumor model 2. The apparent rate constant describing the conversion of Pyr to Lac (*k*_P_) and the [1‐^13^C]Lac/[1‐^13^C]Pyr signal ratios at 30 s were calculated. Previous studies in this tumor model have shown that there is substantial labeling of both the Pyr and Lac pools at 30 s after Pyr injection 2. The repeatability of measurements made 4 h apart is shown in Fig. 2 and Table 1. Non‐fasted animals showed poorer correlations and higher mean differences between successive measurements than fasted animals. Repeatability was also assessed by calculating the COV for the mean *k*_P_ and mean [1‐^13^C]Lac/[1‐^13^C]Pyr signal ratio using data from both time points, i.e. a total of 16 measurements for each group of animals (Table 1). Animals in the fasted state showed lower intra‐individual variability than those in the non‐fasted state, with significantly lower COV for the mean *k*_P_ (*p* = 0.01). The higher metabolic rate of mice *versus* humans means that 4 h is considered to be a substantial fasting period for these animals and equivalent to an overnight fast in humans. Moreover, in the 4‐h period between the MRI examinations, the mice were expected to feed multiple times 24.

The mean *k*_P_ and [1‐^13^C]Lac/[1‐^13^C]Pyr signal ratio calculated from the first and second examinations showed no evidence of a difference for both fasted (*n* = 8) and non‐fasted (*n* = 8) animals. However, when using data from all the mice (*n* = 30), a higher mean *k*_P_ and [1‐^13^C]Lac/[1‐^13^C]Pyr signal ratio were observed in fasted mice than in non‐fasted mice, with the difference in the mean [1‐^13^C]Lac/[1‐^13^C]Pyr signal ratio observed between the first measurements in the fasted (*n* = 11) and non‐fasted (*n* = 17) cohorts achieving statistical significance (*p* = 0.02) (Table 1).

### DCE‐MRI

As both the [1‐^13^C]Lac/[1‐^13^C]Pyr signal ratio and *k*_P_ may be dependent on Pyr delivery 12, tumor perfusion was assessed using DCE‐MRI. There was considerable variation in contrast enhancement between the different animals, as assessed from the ratio of the area under the contrast agent uptake curves at 45 s for the tumor and adjacent muscle. There was no evidence of perfusion changes between fasted and non‐fasted mice at the two time points (corresponding to the two measurements with hyperpolarized [1‐^13^C]Pyr) (Table 2).

### Measurement of metabolite concentrations in tissue extracts

Tissue Lac concentration can affect ^13^C label exchange following the injection of hyperpolarized [1‐^13^C]Pyr and, consequently, the [1‐^13^C]Lac/[1‐^13^C]Pyr signal ratio and *k*_P_ 2, 25. A significantly lower tumor Lac concentration was observed in non‐fasted than in fasted mice after the first Pyr injection (Table 3). There was no evidence of differences in tumor alanine concentrations (Table 3) or in blood Lac levels between fasted and non‐fasted mice (Table 3). The blood Pyr concentration was significantly higher in fasted mice after the first Pyr injection relative to non‐fasted animals (Table 3). There was no evidence of a difference between the groups in terms of tumor weight; however, there was a significant difference in animal weight between fasted and non‐fasted animals at baseline (Group 0) (Table 3).

## Discussion

The repeatability and variability of measurements with hyperpolarized [1‐^13^C]Pyr were determined in fasted and non‐fasted EL4 tumor‐bearing mice. The initial values for the [1‐^13^C]Lac/[1‐^13^C]Pyr signal ratio and *k*_P_ were more similar to the values measured 4 h later in fasted mice than in non‐fasted animals (Fig. 2), giving higher concordance coefficients in fasted animals (Table 1). Both parameters showed higher COVs in non‐fasted mice (Table 1), where the unpredictable dietary intake before and between examinations may have contributed to the higher intra‐individual variability in this group. The lower COV in fasted mice (Table 1) is within a range similar to that seen with other clinical imaging techniques, such as FDG‐PET 26.

There was a significantly higher mean [1‐^13^C]Lac/[1‐^13^C]Pyr signal ratio in fasted than non‐fasted animals at the first time point [5.49 ± 2.23 (*n* = 11) *versus* 3.67 ± 1.70 (*n* = 17), *p* = 0.02] (Table 1) and a significantly higher tumor Lac concentration [19.1 ± 2.7 *versus* 10.8 ± 4.1 (*n* = 3); *p* < 0.05] at this time point (Table 3). This is consistent with previous *in vitro* and *in vivo* studies 2, 25, 27, which have shown that the exchange rate increases with an increase in Lac concentration. There was no evidence for a difference in tumor perfusion between fasted and non‐fasted animals or between the first and second scans in these animals (Table 2), although the error in these measurements was large. Therefore, the higher tumor Lac levels in fasted mice implies that these may have been caused by an increase in tumor glycolysis. This has been observed previously in tumors of fasting animals, where the tumor preferentially consumes glucose in comparison with other tissues and produces increased amounts of Lac 28. The fasted animals also showed higher blood Pyr levels after the first injection of [1‐^13^C]Pyr. This may be explained by increased gluconeogenesis, which results in increased levels of blood Pyr and alanine 29, 30, and occurs independently of the presence of a tumor 31, 32.

The variability of hyperpolarized [1‐^13^C]Pyr metabolism may depend on the organ and/or type and grade of tumor being assessed. Hyperpolarized [1‐^13^C]Pyr was found to give reproducible results in the assessment of normal kidneys of non‐fasted rats; however, the fasted state was not examined in this study 33.

In conclusion, if hyperpolarized [1‐^13^C]Pyr is to be used in the clinic, especially for monitoring response to therapy or disease progression using serial measurements, a knowledge of repeatability is essential. The results presented here suggest that, similar to what is already performed in some clinical centers for ^18^F‐FDG‐PET examinations 16, 17, 34, fasting prior to the MR examination might be preferred because of the more uniform and constant metabolic status and lower intra‐subject variability.

## Supporting information

The raw data acquired during this study and on which the results presented in this paper are based can be found at http://content.cruk.cam.ac.uk/kblab/nbm3568.zip.

Supporting info item
