# Supplementary material for: Effects of fasting on serial measurements of hyperpolarized [1‐13C]pyruvate metabolism in tumors
Source: NMR Biomed. 2016 Jun 16;29(8):1048–55. doi: 10.1002/nbm.3568 (PMC4973679; doi:10.1002/nbm.3568)
Supplement: Supplementary file 1 — Supporting info item [file NBM-29-1048-s001.docx]

Effects of fasting on serial measurements of hyperpolarized

[1-^13^C]pyruvate metabolism in tumors

Eva M. Serrao, Tiago B. Rodrigues, Ferdia A. Gallagher, Mikko I. Kettunen,

Brett W.C. Kennedy, Sarah L. Vowler, Keith A. Burling, and Kevin M. Brindle^1,2^†

**Supplementary Table 1.** Limits of agreement

| Fasting Status | Measure | Mean Difference | Lower 95% LOA (95% CI) | Upper 95% LOA (95% CI) |
| --- | --- | --- | --- | --- |
| Fasted | κ*_P_* | 0.00713 s^-1^ | -0.04330  (-0.07390, -0.01280) | 0.05760  (0.02710, 0.08810) |
|  | Lac/Pyr | 0.479 | -2.710  (-4.640, -0.782) | 3.670  (1.740, 5.600) |
| Non-fasted | κ*_P_* | 0.0132 s^-1^ | -0.0483  (-0.0854, -0.0111) | 0.0748  (0.0376, 0.1120) |
|  | Lac/Pyr | 0.653 | -2.410  (-4.260, -0.558) | 3.720  (1.860, 5.570) |

The mean difference in κ*_P_* between the two scans for the fasted animals is 0.00713 s^-1^. The 95% Limits of Agreement for this difference are -0.0433 (95% confidence interval (CI): -0.0739, -0.0128) and 0.0576 (95% CI: 0.0271, 0.0881). On average the difference in κ*_P_* between the two scans is 0.00713 (i.e. on average κ*_P_* 2 is 0.00713 s^-1^ higher than κ*_P_* 1). Most differences in κ*_P_* between the two scans will lie in the range -0.0433 to 0.0576 (i.e. somewhere between κ*_P_* 1 being 0.0433 s^-1^ more than *k_P_*2 and κ*_P_* 2 being 0.0576 s^-1^ more than *k_P_*1). Similar interpretations apply for the other sets of results.
